# Bioavailability Enhancement and Formulation Technologies of Oral Mucosal Dosage Forms: A Review

**DOI:** 10.3390/pharmaceutics17020148

**Published:** 2025-01-22

**Authors:** Ildikó Bácskay, Petra Arany, Pálma Fehér, Liza Józsa, Gábor Vasvári, Dániel Nemes, Ágota Pető, Dóra Kósa, Ádám Haimhoffer, Zoltán Ujhelyi, Dávid Sinka

**Affiliations:** Department of Pharmaceutical Technology, Faculty of Pharmacy, University of Debrecen, Nagyerdei körút 98, 4032 Debrecen, Hungary; bacskay.ildiko@pharm.unideb.hu (I.B.); arany.petra@euipar.unideb.hu (P.A.); feher.palma@pharm.unideb.hu (P.F.); jozsa.liza@euipar.unideb.hu (L.J.); vasvari.gabor@pharm.unideb.hu (G.V.); nemes.daniel@pharm.unideb.hu (D.N.); haimhoffer.adam@euipar.unideb.hu (Á.H.); ujhelyi.zoltan@pharm.unideb.hu (Z.U.)

**Keywords:** oral mucosal drug delivery, oral cavity dosage forms, bioavailability enhancement, buccal delivery

## Abstract

The oral mucosa is a versatile surface for drug administration, supporting both local and systemic therapies. Many active substances are effectively absorbed in the oral cavity, offering an alternative to enteral administration by bypassing the harsh gastrointestinal environment and hepatic first-pass metabolism. This has made oral mucosal drug delivery a growing area of research. Enhancing the bioavailability of active ingredients is a key focus in pharmaceutical technology, especially given the challenges of developing new drugs. Numerous strategies to improve bioavailability are compatible with oral mucosal delivery, with the unique anatomy of the oral cavity enabling specialized applications. A variety of dosage forms tailored for oral mucosal delivery meet therapeutic needs while addressing biopharmaceutical and patient compliance challenges. Proper formulation can achieve controlled release, improved bioavailability, and patient convenience. This review highlights the potential of oral mucosal drug delivery, focusing on bioavailability enhancement methods and the types and production technologies of dosage forms optimized for use in the oral cavity.

## 1. Introduction

Oral medication is considered the primary therapeutic route because of its patient convenience, safety, and simplicity. Manufacturing traditional oral dosage forms can also be considered easy and cost-effective compared to others. However, the environment of the gastrointestinal (GI) tract is challenging for both the pharmacon and drug delivery systems because of the extreme pH conditions, different types of intestinal cells, and the presence of digestive enzymes, food, and intestinal bacteria [1].

Medicines used in the oral cavity are among the oldest therapeutic tools. Originally, they were intended for mostly local treatment, but the oral mucosa provides an excellent absorption surface for a wide variety of active substances, which cause a systemic effect when entering the bloodstream.

Oral mucosal drug delivery dosage forms have most of the advantages of oral administration, and compliance is even higher in the case of some patient groups like children, elderly people, and patients with swallowing difficulties [2].

Because of the numerous advantages and possibilities, most importantly, easy, convenient, and safe use, quick pharmacological responses, and the opportunity to administer a wider range of pharmacons, oral mucosal drug delivery has been an important topic in academic and applied science for years. We found several examples of articles that summarized the results described in this field from a biological–physiological [3,4,5] or technological formulation [6,7,8,9] point of view. The purpose of this review is to briefly present the currently most important technological opportunities, focusing on biopharmaceutics and bioavailability.

## 2. Bioavailability from the Oral Cavity

### 2.1. Oral Cavity and the Oral Mucous Membrane

**Figure 1:**
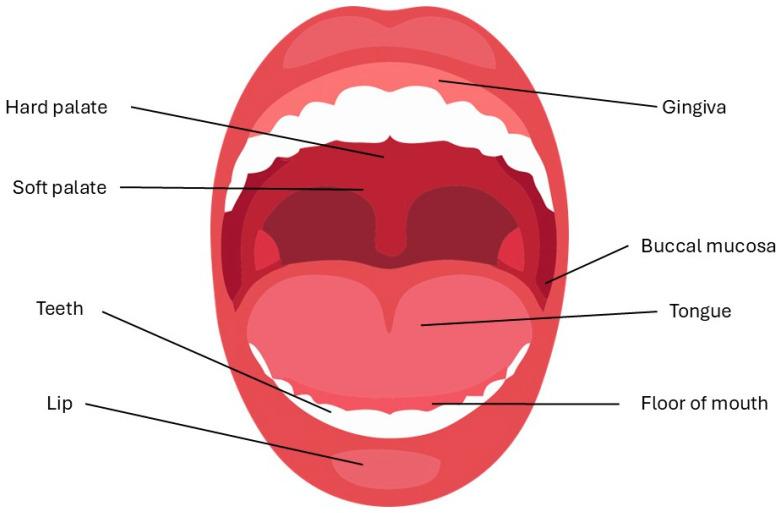
The main anatomical regions of the oral cavity play the most important role in basic functions and medication through the oral mucosa.

The oral cavity is the first receiving part of the gastrointestinal tract. It extends from the mouth opening to the pharynx and is bounded by the cheeks at the side, the floor of the mouth at the bottom, and the hard and soft palates at the top. The teeth are located in the front section, and the tongue is in the bottom (Figure 1) [10]. Several salivary glands are located in the oral cavity, producing saliva and mucus [11]; furthermore, special neuroanatomical structures, namely taste buds, form part of the oral papillae [12].

The mucous membrane or mucosa is a membrane that covers the surface of internal organs and lines the body cavities. It is covered with mucus [13]. The main role of the human body mucosa besides isolation is transport and, to a lesser extent, immunological protection against pathogens. The oral mucous membrane consists of the buccal, sublingual, gingival, palatal, and labial mucosa [14].

The oral cavity, due to its complex anatomical structure, has several functions. The most important is its role in feeding. The food is uptaken, masticated, mixed with saliva, formed into a bolus, and transported to the further parts of the GI tract in the mouth. The digestive process also begins in the oral cavity by the enzymes of the saliva, mainly the amylase, which starts to break down carbohydrates. In addition, being connected to the pharynx, the oral cavity also facilitates breathing. It has an important role in communication, modulating the sound formed in the larynx [15,16,17].

### 2.2. Absorption from the Oral Cavity

Besides the oral drug delivery route, considered the primary route in pharmaceutical development, different mucous membranes of the human body also have significance in pharmacotherapy [18]. The oral cavity is lined by the oral mucosa, which is structurally inhomogenous depending on the function. The upper layer consists of epithelial cells, and the basement membrane, lamina propria, and submucosa are located below that. In these layers, the oral mucosa contains different receptors, like taste buds and smaller salivary glands [19].

Enteral drug delivery is considered the primary route of pharmacon intake, and oral mucosal delivery shares its main advantages. Aside from the high patient compliance, relatively low costs, and administration not requiring special knowledge, it also has additional advantages. These forms of medication are easier to use for patients with swallowing difficulties, but, in many cases, the fact that they do not require liquid for intake is also more convenient [20]. Pharmacons do not degrade in the environment of the GI tract, but by bypassing the hepatic first-pass metabolism, they acquire direct access to the systemic circulation, which means a shorter response time. The oral cavity also allows the use of a wide range of dosage forms [4].

Adequate taste masking and the avoidance of irritation can be considered the biggest challenges in oral mucosal drug delivery. The taste is formed when the substance dissolves in saliva and then contacts the taste buds. Excessive consumption of active substances is toxic to the body in a significant proportion of cases, and the unpleasant bitter taste is an evolutionary defense mechanism against it. The unpleasant taste leads to lower patient compliance and is considered the most important obstacle to therapy in pediatrics. The conventional way to eliminate the problem is taste masking, the sweetening of the preparation with sugar, artificial sweeteners, or various extracts and aromas. Each of these has advantages and disadvantages, such as increased carbohydrate intake or long-term health damage, and is usually used in combination. Modern methods for flavor enhancement include, for example, the use of ion-exchange resin, solid dispersion, spray drying, microencapsulation, and inclusion complexes. However, these technologies are usually more expensive and require more excipients and special formulating conditions. Both approaches (sweeteners and advanced technological taste masking) aim to enhance the palatability of pharmaceutical products by reducement of the sensory impact of bitter drugs and improvement in the patient’s experience. They can be combined for a complementary effect, where sweeteners provide a basic layer of taste masking and eliminate any residual bitterness in tandem with advanced technological methods. The effectiveness of different flavor masking options or their combinations and the specific excipients used can vary from composition to composition, and the subjectivity of taste perception makes their evaluation even more difficult [21,22,23,24].

The volume capacity of the oral cavity (approximately 111 cm^3^ on average [25]) limits the pharmacon dose. Beyond that, the possibility of swallowing, the absorption difficulties due to the physical–chemical properties of the active ingredient, or stability problems are also potential limiting factors [26].

Drug delivery systems administered in the oral cavity can aim for either (a) local or (b) systemic effects; in the latter case, the pharmacon has to be absorbed through the buccal or sublingual mucosa and transported to the submucosa containing blood vessels [27]. The factors affecting absorption primarily are the fluid volume, pH value, enzyme activity, penetration through the mucosa cell barriers, and cell turnover.

Saliva has the most important role in creating the environment of the mouth. It is a thin, hydrophilic liquid secreted by three major and several smaller salivary glands. Saliva lubricates the oral cavity, facilitating swallowing and preventing the demineralization of the teeth. Furthermore, saliva maintains oral enzyme activity, which is part of the digestion, and the oral pH, regulating microbial homeostasis. Saliva production highly depends on several factors, like the time of day or the nature and intensity of stimulation. These factors influence the amount, pH, and composition of the produced saliva [28].

Compared to the orally administered formulations absorbed intestinally, the fluid volume is significantly lower in the mouth, about 1.1 mL, which makes the dissolution and permeation difficult. In addition, elevated saliva production can lead to “saliva wash out”, a phenomenon related to premature swallowing of the product, resulting in inadequate pharmacon liberation and absorption. On the other hand, saliva is less viscous compared to the other gastrointestinal fluids; it contains organic and inorganic components only in 1% of them. The pH value is between 5.5 and 7, which is a much more friendly and steady environment than the more extreme ranges in the further parts of the GI tract. The enzymatic breakdown of drugs is an important problem regarding the whole digestive system, but the enzyme activity of the saliva is much lower than other secretions. This can be of particular importance in the delivery of peptide-type active ingredients since mostly carbohydrates are digested in the oral cavity, and proteases are less present [29,30,31].

The parameters of different GI secretions are compared in Table 1.

The mucus of the oral mucosa consists mainly of proteins and carbohydrates. Its most important macromolecular component is mucin, a glycoprotein capable of forming a network structure and bonding to the surface of the epithelial cells as a gel layer, exerting a lubricating effect [35].

The thickness and structure of the oral mucosa vary, strongly depending on the function of the given part of the oral cavity. The epithelia is keratinized where it is more exposed to mechanical effects, which means better barrier function at the same time. Epithelia contain different lipids, which make them less permeable to water. The buccal and sublingual mucosa are non-keratinized; therefore, the absorption from it is significantly better than from the rest of the oral cavity and is many times that of the absorption from the skin, as can be seen in Table 2 [36].

Both keratinized and non-keratinized mucosa contain membrane-coating granules, which empty their content into the intercellular space to ensure epithelial cohesion, which is a barrier to most penetrating substances [38]. Absorption is better in cell cultures that do not contain membrane-coating granules [39].

Permeation through the mucous membrane can occur by passive diffusion, carrier-mediated active transport, or other special methods like facilitated diffusion or specialized transport mechanisms. According to studies, absorption from the oral cavity is mainly a passive process, paracellular or transcellular. The affected spaces, namely the paracellular space and the cytoplasm, are hydrophilic media, and this favors water-soluble drugs over lipophilic ones; meanwhile, the lipophilic cell membranes of the transcellular route are more passable for apolar molecules [40]. Several studies have described carrier-mediated-active transport of some pharmacons through the oral mucosa [41,42]. In this case, the mediating carriers can be uptake or efflux transporters, proteins located on the apical surface of the epithelial cells. They require energy from ATP breakdown or are paired with a co-transport mechanism of a counterion [43].

Cell replenishment of the oral mucosa is 14–21 days, a shorter period than the 20–30 days cell turnover of the skin [44] because of the high functional demand in the oral cavity. This factor can also play a role in absorption from the mouth [13].

### 2.3. Bioavailability Enhancement Through the Oral Mucosa

There are several options for bioavailability enhancement from the oral cavity; some of them are used in marketed products, and other methods are frequent topics of research (Table 3 and Table 4).

Speaking of bioavailability enhancement on mucous membranes, it is worth mentioning the phenomenon of mucoadhesion, as well as the formulations that take advantage of this. During mucoadhesion, attractive forces are established between a biological material and the mucous membrane or mucus. In the first contact stage, contact occurs at the border of the mucoadhesive and the mucosa. In the second consolidation stage, different interactions take place, strengthening the joint and prolonging the adhesion [69]. There are several theories to describe the process of mucoadhesion, among which the adhesion theory and the diffusion-interlocking theory can be highlighted. According to the adhesion theory, the connection between the adhesive polymer and the mucosa is provided by primary and secondary bonds. The chemisorption is based on ionic, covalent, and metallic bonds [70]. According to diffusion-interlocking theory, a two-way interdiffusion occurs between the mucoadhesive polymer chains and the glycoprotein network of the mucosa [71].

Mucoadhesive drug delivery systems are based on hydrophilic polymer macromolecules. Their essential properties are their negative charge potential and hydrogen bond-forming groups in their structure. Hydroxyl, carboxyl, and amine groups are the most favorable. Adhesion is formed by wetting [72]. Mucoadhesive polymers are divided into two generations [73]. First-generation mucoadhesives are either anionic, cationic, or non-ionic materials. Anionic polymers are the most widely used of them, like polyacrylic acid [74] or sodium carboxymethyl-cellulose [75]. The most common of the cationic substances is chitosan, which can be produced by chitin deacetylation. Chitosan has good biocompatibility and biodegradability properties and can be easily modified by adding different chemical groups into the form that best suits the purpose of use [76]. However, chitosan has the disadvantages of batch-to-batch variability, stability problems, immunogenic and allergenic potential, environmental sensitivity, and the possibility of interactions with drugs [77]. First-generation mucoadhesive polymers share the benefits of easy use and low toxicity [36].

Second-generation mucoadhesive materials are also commonly called cytoadhesive polymers because they bind directly to the surface of the mucous membrane, enabling more precise drug delivery due to the different carbohydrate and protein compositions of the mucous membrane in different areas. Lectins are natural polymers that have a basic role in biological recognition. After binding to the mucosa, lectins can remain on the cell surface, or in the case of receptor-mediated adhesion, they can be internalized by endocytosis. Although lectins have advantages in terms of both targeting and binding, their use is limited by their toxic or immunogenic properties, in addition to the ability of lectin-induced antibodies to interfere with mucoadhesion [78]. Thiomers are thiolated polymers produced via the modification of materials like polyacrylates, chitosan, or deacetylated gellan gum. Thiol groups form covalent bonds with the deeper, cysteine-rich layers of mucin. The covalent bonds formed by second-generation mucoadhesives are less sensitive to the changes in ionic strength or pH, although thiol groups can be oxidized at pH values below 5, which can lead to a destabilized thiomer structure [79,80].

The other possibility for increasing bioavailability is the use of penetration enhancers, which are common in a wide range of dosage forms. Drug permeability from the oral mucosa can also be increased with these excipients, such as bile salts, like sodium deoxycholate or sodium taurocholate; fatty acids, namely sodium laurate, sodium myristate, and oleic acid; and surfactants, namely sucrose laurate, cyclodextrins, chelators, ethanol, propylene glycol, and chitosan, which are the most commonly used materials [81].

In the case of some formulations, bioavailability enhancement is achieved by the possibly fastest disintegration of the product and almost immediate liberation of the pharmacon, often in a few seconds after administration, in contrast to the prolonged retention time of mucoadhesive systems. All this is in the relatively low liquid volume characteristic of the oral cavity [82].

Although enzyme activity is significantly lower in the saliva than in the other parts of the GI tract, it still can damage the active ingredients. Pharmacon degradation can be decreased by the incorporation of enzyme inhibitor components, with aprotinin, bestatin, and puromycin being the most commonly used [83,84]. Protease inhibition also enhances the absorption and bioavailability of peptide and protein-type drugs, which is a considerable advantage of oral mucosal drug delivery over enteral drug administration [85].

There are several possibilities to modify active ingredients to increase their bioavailability. Prodrugs are inactive forms of the pharmacon that transform to the parent drug during absorption or metabolism [86]. The buccal bioavailability of morphine proved to be increased in vivo by the use of morphine-esther prodrugs [55]. Particle size reduction also enhances solubility and permeability in general, and studies can be found of buccal bioavailability increases with this method for different active ingredients [87,88]. By modifying the lipophilicity of the molecules, the permeation through the lipid-rich membrane of the mucosa can be improved [89].

Different carrier formulations like nanoparticles and liposomes encapsulate drug molecules, protecting them and providing increased absorption. In addition, they can achieve controlled, prolonged release of the active ingredient, which can further improve bioavailability [90]. Pullulan-based particles were found to have a long retention time and sustained pharmacon release profile, leading to better delivery to less accessible regions, as well as reduced irritation and improved patient compliance [91].

Micro- and nanosized particles enable faster and increased absorption from the oral mucosa. The various types of particles have different formulation methods and advantages. Liposomes improve the bioavailability of pharmacons with poor water solubility and mucosal permeability from solid forms and also enhance the stability of active ingredients, which is an important advantage in phytopharmacon therapy [65]. Microparticles based on suitable polymers have mucoadhesive properties, elongating the retention time on the surface of the oral mucosa [92]. The proper polymer blend of polyglycolic acid derivatives shows suitable adhesive properties not only to the mucosa but also to the dental surface, which leads to an elongated and more effective antimicrobial treatment [93]. Permeability improvement and enhanced systemic effects can be achieved with solid lipid nanoparticles [94] or polystyrene nanoparticles [95], which are absorbed through the mucous membrane cells.

Fast-dissolving particles formulated by freeze-drying [96], possibly combined and stabilized with beta-cyclodextrins [97], are able to significantly increase solubility (even with the minimal amount of fluid in the oral cavity) and, therefore, plasma concentration.

Micro- and nanoparticles can be incorporated into most of the dosage forms intended for oral mucosal drug delivery. Aqueous suspensions, aerosols, or ointments are the easiest ways to use them. Their small size results in more convenient use for the patient, but on the other hand, the lower predictability than unit dose forms, in terms of the administered pharmacon amount, can be a disadvantage.

## 3. Oral Mucosal Drug Delivery Dosage Forms and Formulation Technologies

### 3.1. Liquid

Liquid dosage forms applied in the oral cavity are typically solutions or suspensions in a proper (aqueous) vehicle. Their use is determined basically by their short retention time on the oral mucosa. Also, targeted delivery is often problematic to achieve, and absorption is difficult to control. Accordingly, the products are primarily antibacterial mouthwashes and mouth fresheners intended for local use, like Listerine or Curasept [98,99].

Regarding systemic therapy, with a significantly different and wider range of treatment possibilities, oral liquid drug delivery systems (OLDDSs) and oral liquid controlled release (OLCR) have gained attention in recent years because of their numerous advantages, like their easy administration and good spreadability [100]. Iontophoretic technology is a promising method for improving absorption and systemic treatment. Iontophoresis is based on electrophoresis, electroosmosis, and electropermeabilization and is found to be safe and effective in the case of buccal prilocaine, lidocaine, naltrexone, sumatriptan, and atenolol delivery, among other active ingredients [101].

### 3.2. Semi-Solid

The semi-solid dosage forms used in the oral cavity include gels, creams, and ointments, which are applied topically to exert a local or systemic effect. The base of the formulation is usually some type of polymer. The most obvious is the use of hydrogels since polymers that can hydrate in the aqueous environment of the oral cavity can trap the active ingredient and prolong its release during diffusion or erosion. Semi-solid formulations can be applied with the fingers or a suitable application device, which can facilitate placement in the right spot and easy spreading on the mucous membrane. In many cases, their use means a better feeling for the patient compared to solid drug forms [102]. At the same time, the amount of pharmacon delivered is in a much wider range compared to unit dose formulations, and semi-solid systems spend a short retention time in the oral cavity. The latter problem can be eliminated by using bioadhesive polymers in the composition [103]. The commercially available products are mostly local analgesics like Xylonor or mouth moisturizers like OralBalance.

### 3.3. Solid

Solid dosage forms are the most common, considering their advantages in terms of production, stability, and application. They are also the most diverse group of oral mucosal medications regarding formulation technology methods.

**Medical lozenges** are flavored, often sugar-based formulations designed to slow disintegration in the oral cavity. The pharmacon is released directly to the mucosa of the mouth and the throat from the lozenges, which can be divided into hard and soft lozenges. Medical lozenge formulation technologies include melting and direct compression. Their key characteristic is their slow pharmacon release. Among the disadvantages of medical lozenges, we can find the danger of overuse, especially for children, as well as risks regarding premature swallowing in the case of some formulations. Furthermore, lozenges generally spend circa 30 min at most in the oral cavity, which limits the incorporated pharmacon quantity, and the disintegration depends on the patient (increased saliva production, chewing). Hard and soft lozenges are among the oldest forms of medicine used in the oral cavity, primarily for local therapy, mostly for sore throats, local anesthesia, and disinfection; less often with pharmacons that enter the systemic circulation, such as nicotine, cough suppressants, and vitamin preparations. The primary methods for the formulation of medical lozenges are direct compression or melting and molding processes. During direct compression, the active ingredients and auxiliary materials, including polymers and sweeteners in the form of powder or granules, are pressed directly. Melting and molding are based on the precise temperature control of the heated base materials, mostly polymers, sugar, and binding agents, forming a homogenous mass. Then, the APIs are added, and the liquid is poured into a properly designed mold, ensuring precise dosing and consistency in distribution. In the mold, the lozenges are cooled to solidify at room temperature or in refrigerated chambers [104]. They are not the focus of modern drug delivery system research despite the fact that studies found them a promising dosage form in the bioavailability enhancement of drugs like resveratrol and also in combination technology with self-emulsifying systems [105,106,107,108]. Several popular medical lozenges are available commercially, like the antiseptic Strepsils or the smoking cessation aid Nicorette.

**Medicated chewing gum** is an extended-release dosage form with similarities to lozenges. The main difference is that chewing gums contain a water-insoluble base like latex, plastics, solid paraffin, or bees’ wax, which is not dissolved during the application. They are manufactured by direct compression or fusion. Chewing gum products are most commonly used in therapies for motion sickness (Travelgum), smoking (Nicorette), pain (Aspergum), and nausea (Biodramina) [109]. Unlike lozenges, chewing gum can cause stress on the jaws, leading to joint and muscle disorders in the long run. Also, they require a wider selection of different excipient types for proper formulation, sometimes pairing with more complicated manufacturing methods, although the most commonly used techniques are direct compression and melting and molding [110].

For the formulation of **buccal matrix tablets**, a bioadhesive polymer, like carbopol, hydroxypropyl methylcellulose, modified starch, sodium alginate, or polyacrylic acid, is incorporated into the matrix containing the pharmacon and the excipients. The tablet adheres to the mucous membrane of the oral cavity and, in accordance with the general properties of matrix tablets, enables a prolonged release of the active ingredient [111]. Matrix tablet structures can be various; by incorporating an inert or impermeable layer, the release and loss of the active ingredient in the direction of the oral cavity can be prevented, allowing only pharmacon release towards the mucous membrane. Buccal mucoadhesive matrix tablets can be a proper vehicle system for different types of drugs, including peptides such as insulin, calcitonin, and Glucagon-like peptide 1 [112]. A further advantage of matrix tablets is that they can be formulated using the equipment for traditional tablet manufacturing by methods like direct compression [113] or dry granulation [114]; the function of the preparation is based on the selection of excipients. Striant is a buccal matrix system designed for controlled and sustained testosterone delivery.

**Orodispersible tablets (ODT)** are based on a porous or soft matrix. This technology, in contrast to other solid forms of oral mucosal drug delivery, targets rapid disintegration and dissolution in the mouth; therefore, the goal during production is to maximize the porosity of the tablet matrix. The excipients used during the formulation can promote disintegration and increase or accelerate water solubility. By these methods, we can ensure the rapid penetration of water into the tablet matrix, the short disintegration time of the system, and the quick dissolution of the active ingredient. Moreover, the formulation should provide this without requiring water for tablet intake. Co-processed excipients are widely used for the formulation of ODTs. They are combinations of two or more excipients designed to enhance physical properties, mostly flowability, compressibility, and rapid disintegration. They are produced by spray drying, wet granulation, or co-crystallization. The most popular products are F-Melt^®^ (Fuji Chemical Industries Co., Ltd., Toyama, Japan) (carbohydrates and disintegrants), Ludiflash^®^ (BASF Pharma, Ludwigshafen, Germany) (mannitol, crospovidone, and polyvinyl acetate), StarLac^®^ (MEGGLE Pharma, Wasserburg am Inn, Germany) (lactose and starch), Prosolv^®^ (JRS PHARMA, Rosenberg, Germany) (microcrystalline cellulose and colloidal silicon dioxide), and Parteck^®^ variations (Merck KGaA, Darmstadt, Germany) [115]. ODTs are beneficial for patient groups with swallowing difficulties, and the risk of suffocation can also be avoided with them. At the same time, a disadvantage is their hygroscopic nature, which may require special packaging, as well as the slightly more complicated and expensive technology involved in their production [116,117,118].

**Figure 2:**
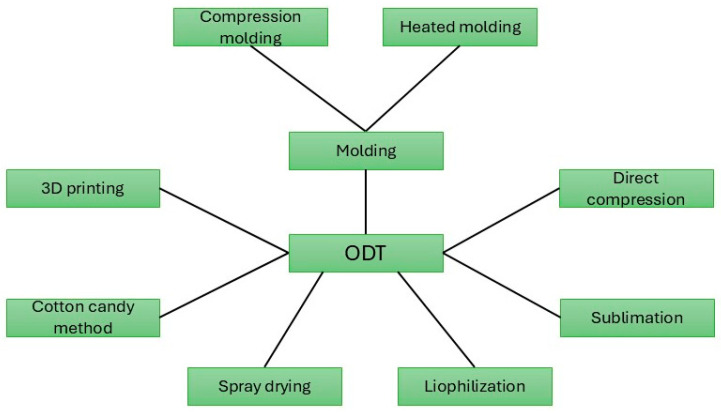
Visual overview of the formulation possibilities of ODT preparations. The various technological processes ensure the fastest disintegration in the minimum liquid medium.

There are several technological options for the production of ODTs, as can be seen in Figure 2, of which the direct pressing process is the most simple and economical, similar to traditional tablets. The selection of excipients that ensure the appropriate porosity is crucial. The most commonly used disintegrant materials are different types of starch or starch derivatives, like sodium starch glycolate and pregelatinized starch. Microcrystalline cellulose and mannitol are also often used [10,119,120].

The porosity of the tablets produced by direct pressing can be further increased by **sublimation** technology. In this process, volatile components (e.g., ammonium carbonate, ammonium bicarbonate, camphor, benzoic acid, naphthalene, urea, urethane, thymol, phthalic anhydride) are incorporated into the matrix [121]. These additives sublimate later, leaving numerous pores and channels in the tablet. Through these openings, the solvent can more easily and rapidly enter the tablet, and the disintegration time can be shortened to 10–20 s [122]. Nimulid-MD for pain and inflammation is manufactured using this method.

During **lyophilization**, in the first step, the temperature of the solvent is decreased below the freezing point, and the system solidifies. Then, in the second step, the solvent sublimates from the system at low pressure and increasing temperature. The remaining solid material has an amorphous structure and is extremely porous, which makes its rapid dissolution possible [123,124]. The pharmacon can also be placed into an aqueous solution of the carrier system in the prepared blister pack. The mixture freezes while passing through a liquid nitrogen tunnel. After rapid and efficient low-temperature freezing, the blisters are freeze-dried at low pressure, and then the packaging is closed after the solvent has evaporated [81]. The technology is expensive, but it makes the incorporation of heat-sensitive substances possible [125]. The formulation of Claritin RediTabs, an antiallergic medication based on Zydis Technology, using the liophilization method.

Two types of **molding methods** are distinguished. In compression molding, the powder mixture is placed in a water–alcohol solvent, and this wet mass is pressed into a mold under low pressure. Then, the solvent is air-dried, and the remaining tablet is solid and porous. During the heated molding method, a suspension made from a mixture of the active ingredient, some type of sugar (e.g., mannitol, sorbitol, lactose), and agar is poured into the cavities of the prepared blister pack, where the agar solidifies and forms a gel structure. The product is dried under vacuum [126]. Molding is used in the formulation of Panadol ActiFast ODT for pain relief and fever reduction.

**Spray drying** breaks down the solution or suspension into small droplets using a spray head, increasing the surface area so that the solvent can be quickly removed in the flow of drying air, and the resulting steam forms a protective layer around the particles. Heat-sensitive materials can also be processed with this technology. The dried particles are porous and suitable for compression. Formulations with spray-dried excipient bases have proven to have enhanced drug release in comparison with directly compressed products, such as valdecoxib and metoclopramide [127]. The spray drying technique is used for the production of Maxalt-MLT for acute migraine treatment.

**The cotton candy method** is based on saccharides and polysaccharides (e.g., polymaltodextrin and polydextrose) that are quickly melted at 180–260 °C and formed into sugar fibers using a spin-like device. Then, the fibers are ground and mixed with active ingredients and excipients. This mixture is pressed into a tablet, resulting in appropriate mechanical and structural characteristics [128]. FlashDose technology incorporates the cotton candy method and is used for the production of Nurofen Meltlet, a fast-acting pain relief.

It is possible to create a porous tablet matrix by building layers of the product on top of each other using **3D printing** technology. The tablets produced this way disintegrate in the oral cavity in a few seconds, but their production is slow and expensive compared to other technologies. 3D-printed tablets containing various active ingredients are heavily researched, and the results of technologies like selective laser sintering and fused deposition modeling are promising. Inside the selective laser sintering machine, a thin layer of powder is spread. A computer-controlled laser selectively scans it, heating specific areas to a temperature where the particles melt together. After one layer is completed, the building platform slightly descends, and a new layer of powder is spread. The process is repeated layer by layer until the entire product is formed. Fused deposition modeling is also based on layer-by-layer printing on a descending platform, but this technology uses a filament containing APIs fed into a heated nozzle, which melts and extrudes it on the building platform according to the digital design [129,130]. Only one marketed 3D-printed ODT product can be found, Spritam, for the treatment of epilepsy, using ZipDose technology [131].

Bioadhesive **buccal discs** are similar to tablets in numerous properties but thinner and flatter than others, and their shape can be adjusted according to the application in the buccal cavity. Based on the tests, they are less irritating, easier and more comfortable to use, and do not cause a dry mouth [132,133]. The easiest way for the formulation of buccal discs is direct compression [134]. There are fewer buccal disc products on the market; some examples of them are OraMoist and XyliMelts.

### 3.4. Spray

Aerosol sprays are able to distribute the active ingredient evenly in the saliva or on the surface of the mucous membrane. Their use is convenient and simple for the patient, and the small droplets or particles can reduce the absorption time [135]. This possibility plays a major role in researching the administration of protein- and peptide-type active substances as an alternative to injections. Buccal insulin spray, absorbed from the oral mucosa, produces a plasma concentration similar to subcutaneous insulin pens [136], and its absorption can be improved with various excipients, like soy lecithin or propanediol [137]. Currently, several buccal sprays are in development, based on the RapidMist technology, with various active ingredients, including vaccines. The aim of this formulation technology is to deliver liquid medicines into the mouth precisely and in exact quantities. The pharmacon is encapsulated in micelles of penetration enhancers and delivered in a high-velocity fine particle aerosol that spreads on the surface and traverses to the bloodstream through the mucosa, appearing in the circulation within 10 min after administration [138]. Different types of buccal sprays are available, varying from local antibacterial preparations (Cepacol) to systemic nitroglycerin (Nitrolingual Pumpspray) and zolmitriptan (Zomig) medicines.

### 3.5. Films and Patches

The oral cavity mucous membrane is an excellent surface for modified drug release systems, as it is an extensive and immobile tissue. Mucoadhesive patches can be used to achieve a local or systemic effect. Release of the active ingredient is prolonged from them, and even with a lower active ingredient content, they provide a high plasma level curve and a stable pharmacon release (even hours later), compared to gels that wash off faster. Preparations on the market contain buprenorphine (Belbuca) and fentanyl (Onsolis), among others. There are ongoing experiments of immunization through buccal films to replace injections [139].

These dosage forms are typically made by pouring a polymer solution, active ingredient, and excipients onto an appropriate surface and then drying it. Their size can vary widely, but a surface of 1–3 cm^2^ is ideal for application. In terms of pharmacon release, multidirectional and unidirectional preparations are distinguished; in the latter case, the polymer layer is applied to an impermeable background layer. Compared to buccal tablets, the films and patches are thinner and more flexible. This makes their use easier and more comfortable for the patient, but at the same time, the risks of damage, overhydration, and loss of adhesive properties of the product are also elevated [140].

The solvent evaporation process is the primary method for production. The polymer is dissolved in a solvent or solvent mixture, and then the active ingredient is dissolved or dispersed in the liquid. It is then transferred to a suitable surface from which the solvent can evaporate, leaving behind a solid polymer film. Direct pressing and hot-melt extrusion are also popular production methods because of their avoidance of organic solvent use [141,142]. During hot-melt extrusion (HME), raw materials (active ingredients, polymers, excipients, etc.) are fed into the extrusion equipment, typically in a powder or granular form. Inside the extruder, the materials are subjected to controlled heat and shear force. This melts and homogenizes the mixture, creating a uniform mass. The molten mass is forced through a shaped die to produce the desired form. Then, the extruded material is cooled to solidify into its final form. Lastly, the extruded product is cut or shaped. Aside from the films, HME is a method for the production of pellets, granules, tablets, and solid dispersions and can achieve controlled release and taste masking [143].

## 4. Conclusions

Looking at the scientific literature of recent years, it can be seen that oral mucosal drug delivery has serious potential. Its many advantages include convenient and simple use, versatility, modern technological options, and efficiency.

It can be seen that the trends are diverse within the field. The two main options for effective drug delivery are the longest possible retention time on the mucous membrane and, on the contrary, the fastest possible disintegration and active substance release. In the first case, the most important technological challenge is the selection of the right mucoadhesive polymer, which is biocompatible, leaves no residue or unpleasant feeling in the oral cavity, and provides a suitable controlled release of the active ingredient, which lasts sufficiently but not too long. The most frequently investigated mucoadhesive dosage forms are gels, films, and matrix tablets.

In the other case, the most difficult task is ensuring the relatively fast disintegration, often less than a minute, in a minimal amount of liquid. For this, a suitable disintegrant auxiliary material must be chosen, or an extremely porous structure must be created during the formulation process, all in balance, with sufficient stability before application. In this field, ODT tablets are the most popular, but their production requires careful planning and, in some cases, a more complicated technological process than is usual for oral tablets.

Both the longer retention time and rapid disintegration can be combined with technologies that increase the absorption of the active ingredient; in both cases, they lead to bioavailability enhancement.

Looking at the prevalence of oral mucosal drug delivery dosage forms, two trends can also be observed. The focus of scientific research is on more modern, complex drug delivery systems, such as nanoparticles or liposomes. In the case of products intended for commercial use, preference is given to formulations that can be developed and produced in a more cheap and simple way. Medicated jelly gums and lozenges can be considered a good example, as they are underrepresented in modern research compared to other forms of oral medicine, but at the same time, there is an increasing number of these types of preparations on the market.

Regardless of the pharmaceutical form, it is essential to create the right taste for all preparations used in the oral cavity. This can also be a challenge since the size of the carrier system limits the amount of excipients that can be used, while the typically bitter taste of the active ingredients requires a large amount of flavor enhancers. The subjectivity of taste perception also makes testing difficult. Determination of the appropriate taste effect in the planning phase is hard, even though the success of the commercial product and the therapy is decisively determined by this.

It is likely that these trends will converge in the future. As the effectiveness of the researched modern and complex pharmaceutical forms increases and their production becomes simpler, such preparations will be able to be marketed in a greater proportion. At the same time, the market can also have an impact on scientific research, and simpler, more commercially successful forms of medicine can also become more popular for researchers. The importance of oral drug delivery is indisputable, considering the number of scientific publications on the subject and patient compliance. An excellent example of the possibilities of the modern approach is that models of the oral mucosa have been developed to replace animal experiments, making research more ethical and taking sustainability into account [144].

The increasing number of peptide-type pharmacons will probably make this route of administration even more important as an option to replace injections. Formulation solutions and methods of increasing bioavailability are currently diverse and are also the focus of research.

The future of oral mucosal drug delivery systems is likely to experience an increasing number of products made with different manufacturing technologies on the market, the expansion of ranges of active substances that can be used in this route due to various bioavailability enhancement methods, and the replacement of invasive intake in the case of biological therapy, vaccination, and peptide-type pharmacons.

## Figures and Tables

**Table 1:** Comparison of different gastrointestinal secretions [32,33,34].

	Volume (Mean)	pH	Enzyme Activity
Saliva	1.1 mL	5.5–7	Low
Gastric fluid	45–686 mL	2.9–5.4	High
Intestinal fluid	54–105 mL	6.8–7.2	High
Rectal fluid	1–3 mL	7–8	Low

**Table 2:** Comparison of permeability from the skin and the oral mucosa regions, adapted from Lesch et al. [37].

Region	Permeability Constant (Kp (×10^−7^ ± SEM cm/min))
Skin	44 ± 4
Hard palate	470 ± 27
Buccal mucosa	579 ± 16
Lateral border of tongue	772 ± 23
Floor of mouth	973 ± 33

**Table 3:** Options for bioavailability enhancement in oral cavity I.

Method	Advantage	Disadvantage	Literature
Mucoadhesion	- Easy use - Low toxicity	- Limited absorption capacity - Circumstances like pH can affect release	[45,46,47]
Penetration enhancement	- Inert, non-toxic materials	- Possible irritation and alteration of stability - Unpleasant sensory properties	[48,49]
Fast disintegration	- Immediate release—easy administration	- Stability concerns - Complicated production	[50,51,52,53]
Enzyme inhibition	- Higher pharmacon stability	- Potential side effects of excipients	[52,53,54]
Prodrug	- Targeted delivery - Improved pharmacokinetics	- Delayed effect and possible toxic metabolites	[55,56,57]
Nanoparticles	- Sustained release - Reduced irritation	- Manufacturing challenges - Possibility of toxicity and bioaccumulation	[58,59,60,61]
Modifying lipophilicity	- Enhanced cellular uptake	- Solubility concerns	[62,63,64]
Liposomes	- Biocompatibility - Enhanced membrane penetration	- Higher production costs - Large-scale production problems - Stability issues	[65,66,67,68]

**Table 4:** Options for bioavailability enhancement in oral cavity II.

Method	Common Excipients	Pharmacon Examples	Literature
Mucoadhesion	Polyacrylic acid, carboxymethyl-cellulose, chitosan	Fentanyl, miconazole, theophylline, dexamethasone	[45,46,47]
Penetration enhancement	Sodium deoxycholate, sodium myristate, cyclodextrin	Insulin, other peptides	[48,49]
Fast disintegration	Microcrystalline cellulose, camphor, mannitol	Propranolol, metformin, ibuprofen	[50,51,52,53]
Enzyme inhibition	Aprotinin, bestatin	Insulin, calcitonin, thymopentin	[52,53,54]
prodrug		isoniazid, levodopa, morphine	[55,56,57]
Nanoparticles	Polystyrene, pullulan, chitosan, hydroxypropyl methylcellulose, oleic acid	Tedizolid, repaglinide, dexamethasone, leuprolide	[58,59,60,61]
Modifying lipophilicity	Polyethylene glycol	Warfarin, naproxen, verapamil, lidocaine, progesterone, cyclosporine	[62,63,64]
Liposomes	Phosphatidylcholine, cholesterol, soybean lecithin	Resveratrol, calcitonin, efavirenz	[65,66,67,68]
